# Incidence of venous thromboembolic events not related to vascular catheters in a prospective cohort of critically ill children

**DOI:** 10.1007/s00431-022-04487-8

**Published:** 2022-06-02

**Authors:** Åsa K. M. Östlund, Urban Fläring, Peter Larsson, Sylvie Kaiser, Lena Vermin, Tony Frisk, Ann Dahlberg, Jonas Berner, Åke Norberg, Andreas Andersson

**Affiliations:** 1 Department of Paediatric Perioperative Medicine and Intensive Care, Astrid Lindgren Children’s Hospital, Karolinska University Hospital, Stockholm, Sweden; 2 Department of Physiology and Pharmacology, Karolinska Institutet, Stockholm, Sweden; 3 Department of Paediatric Radiology, Astrid Lindgren Children’s Hospital, Karolinska University Hospital, Stockholm, Sweden; 4 Department of Children’s Health, Astrid Lindgren Children’s Hospital, Karolinska University Hospital, Stockholm, Sweden; 5 Department of Clinical Science, Intervention and Technology, Karolinska Institutet, Stockholm, Sweden

**Keywords:** Clinical study, Critical care, Heparin, Paediatrics, Venous thrombosis

## Abstract

The risk for venous thromboembolism (VTE) is considered to be low in the general paediatric intensive care unit (PICU) population, and pharmacological thromboprophylaxis is not routinely used. PICU patients considered at high-risk of VTE could possibly benefit from pharmacological thromboprophylaxis, but the incidence of VTE in this group of patients is unclear. This was an observational, prospective study at a tertiary multi-disciplinary paediatric hospital. We used comprehensive ultrasonography screening for VTE in critically ill children with multiple risk factors for VTE. Patients admitted to PICU ≥ 72 h and with ≥ two risk factors for VTE were included. Patients receiving pharmacological thromboprophylaxis during their entire PICU stay were excluded. The primary outcome of the study was VTEs not related to the use of a CVC. Ultrasonography screening of the great veins was performed at PICU discharge. Seventy patients with median (interquartile range) 3 (2–4) risk factors for VTE were evaluated. Median age was 0.3 years (0.03–4.3) and median PICU length of stay 9 days (5–17). Regarding the primary outcome, no symptomatic VTEs occurred and no asymptomatic VTEs were found on ultrasonography screening, resulting in an incidence of VTEs not related to a vascular catheter of 0% (95% CI: 0–5.1%).

*Conclusion*: Our results indicate that VTEs not related to a vascular catheter are a rare event even in a selected group of severely ill small children considered to be at high risk of VTE.

**Table:** 

**What is Known:** *• Children in the PICU often have several risk factors for venous thromboembolism (VTE).* *• The incidence of VTE in PICU patients is highly uncertain, and there are no evidence-based guidelines regarding VTE prophylaxis.*	
**What is New:** *• This study found an incidence of VTEs not related to a vascular catheter of 0% (95% CI: 0–5.1%).* *• This indicates that such VTE events are rare even in PICU patients with multiple risk factors for VTE.*

**Supplementary information:**

The online version contains supplementary material available at 10.1007/s00431-022-04487-8.

## Introduction

Paediatric venous thromboembolism (VTE) carries the risk of significant complications, including sepsis and post-thrombotic syndrome [1]*.* In healthy children, the risk of VTE is low and VTE is mainly considered to be a disease of hospitalized and critically ill children [2]. However, in critically ill children, clinical signs of VTE can easily be missed or ascribed to the underlying condition. This is especially true in small children with limited communication abilities, and where venous collaterals often develop rapidly. Even occlusive VTE can be asymptomatic in children [3]. In order to investigate the true incidence of VTE in paediatric intensive care unit (PICU) patients, screening for VTE is necessary [4].

Even though previous data is limited, VTE seems to be an uncommon event in the general PICU population [5]. This suggests that pharmacological thromboprophylaxis (pTP) might not be indicated in a broad PICU population without specific risk factors for VTE. The evidence-based guidelines on antithrombotic therapy for children by the American College of Chest Physicians do not recommend the universal adoption of pTP in the general PICU population [6]. In line with this, only 4.5% of PICU patients received pTP with low molecular weight heparin (LMWH) in a multinational study of pTP practice in critically ill children published in 2014 [7]. However, most data in the field originate from retrospective studies of symptomatic cases, presumably underestimating the true incidence of VTE. The lack of robust data on which to base guidelines has resulted in a large variation in the routines for prescription of pTP for PICU patients [7; 8]. Moreover, there is a lack of evidence on the efficacy of pTP in children. In adult ICU patients, there is a 50% lower risk of VTE with the use of heparins for VTE prophylaxis [9], but no data from the paediatric setting exist. There are also possible adverse effects of using pTP. Administration of LMWH is painful to children and has potential side-effects such as bleeding [10], heparin-induced thrombocytopenia and neonatal osteopenia [11].

Patients in the PICU are a heterogenous group, and the indication for pTP may be stronger in PICU patients considered to be at high risk of VTE. Recent recommendations suggest that PICU patients with at least two risk factors for VTE may benefit from pTP [11], but there is currently no data describing the incidence of VTE in this population. Such data is necessary to better understand the risk/benefit ratio for pTP in high risk patients, and to adequately power randomized controlled studies investigating the efficacy of pTP in this setting.

In this study, we conducted a comprehensive screening for VTE using doppler ultrasonography (US) in critically ill children admitted to the PICU for ≥ 72 h and with at least two risk factors for VTE. Ultrasound evaluation of the great veins was performed to ensure that also asymptomatic VTEs were included in the study. Our main objective was to provide new data on the incidence of VTE in a group of PICU patients considered to be at high risk of VTE.

## Methods

The Regional Ethics Review Board in Stockholm (reference No 2015/140–31/1, 15/02/2015) approved the study. The study protocol was registered at the Australian New Zeeland Clinical Trial Registry (ACTRN12615000441516). Consent was obtained from parents and when possible from the child.

### Study design

This was a prospective observational study, conducted at the PICU of a multi-disciplinary referral centre for critically ill children in Stockholm, Sweden. Patients were consecutively included from April 2015 to November 2016, although inclusion was paused during 6 weeks in the summers of 2015 and 2016 due to lack of ultrasound resources.

All patients < 18 years of age, weighing ≥ 1250 g, admitted to the PICU for ≥ 72 h, and with at least two risk factors for VTE were eligible for inclusion. Patients were not eligible for inclusion if death was deemed imminent and inevitable.

### Study procedure

Data on patient demographics and risk factors were collected using a standardized report form. Risk factors for VTE included the presence of a CVC, congenital heart disease, trauma, cancer, previous VTE, perioperative patient, renal failure, invasive mechanical ventilation, sepsis, age < 1 year or > 12 years, oral contraception, active inflammatory disease or antiphospholipid syndrome. Sepsis was defined according to international consensus guidelines [12]. Acute kidney injury (AKI) was defined as serum creatinine increased > twofold above either baseline or the upper limit age-adjusted reference intervall, or the need for continuous renal replacement therapy (CRRT). The recorded patient data included age, prematurity (for children < 1 year), gender and body weight. PICU admission diagnosis and length of stay were noted. Pediatric Index of Mortality-2 (PIM-2) score was used to determine degree of illness at PICU admission. The PICU requirement for mechanical ventilation, CRRT and extracorporeal membrane oxygenation (ECMO) was documented. Platelet count, activated partial thromboplastin time (aPTT), international normalized ratio (INR), antithrombin III, fibrinogen level and d-dimer level at admission and discharge were registered if available. Children were not submitted to additional needle-pricks in order to obtain missing coagulation parameters. Patients receiving pTP or anticoagulation therapy during their entire PICU stay (defined as < 48 h without pTP/anticoagulation therapy) were excluded from the study.

### Outcome measures

The incidence of VTEs that were not related to a CVC was chosen as the primary outcome of the study. Patients were followed for clinical signs of VTE (swelling of extremity, pain, discoloration or erythema, superior vena cava syndrome, visible collateral veins) during their PICU stay. An extensive screening for VTE using compression US with colour Doppler was performed at the time of discharge from the PICU. The veins evaluated included lower extremity (popliteal, superficial femoral, common femoral), upper extremity (subclavian, internal jugular, brachiocephalic) and intraabdominal (external iliac, common iliac, inferior caval, portal, renal) veins. The veins with a previous or existing CVC were included in the US screening protocol. US was performed by a paediatric radiologist or sonographer. Linear array vascular transducer of 5–12 MHz and a width of 6–8 cm was used. In most patients, Siemens Acuson S 2000 was used, while a small number of patients were examined with a Philips EPIQ7. The diagnosis of venous thrombosis was made on the basis of previously described direct and indirect signs [13]. Symptomatic VTE was defined as the presence of swelling, pain, discoloration or erythema, visible collateral veins or superior vena cava syndrome. Management strategy and follow-up plan for VTEs were decided by the clinical team managing the patient.

### Statistical analysis

To assess normality, D’Agostino Pearson omnibus normality test was used. Non-parametric data are presented as median and interquartile range (IQR) and normally distributed data as mean with standard deviation (SD) as distribution measure. Wilcoxon signed-rank test was used to compare groups with paired, non-parametric data. Proportions are presented with their 95% confidence intervals (CI). Analyses were performed using GraphPad Prism 8.0.1(GraphPad Software, 2365 Northside Dr, Suite 560 San Diego, CA 92,108, USA).

## Results

**Fig. 1:**
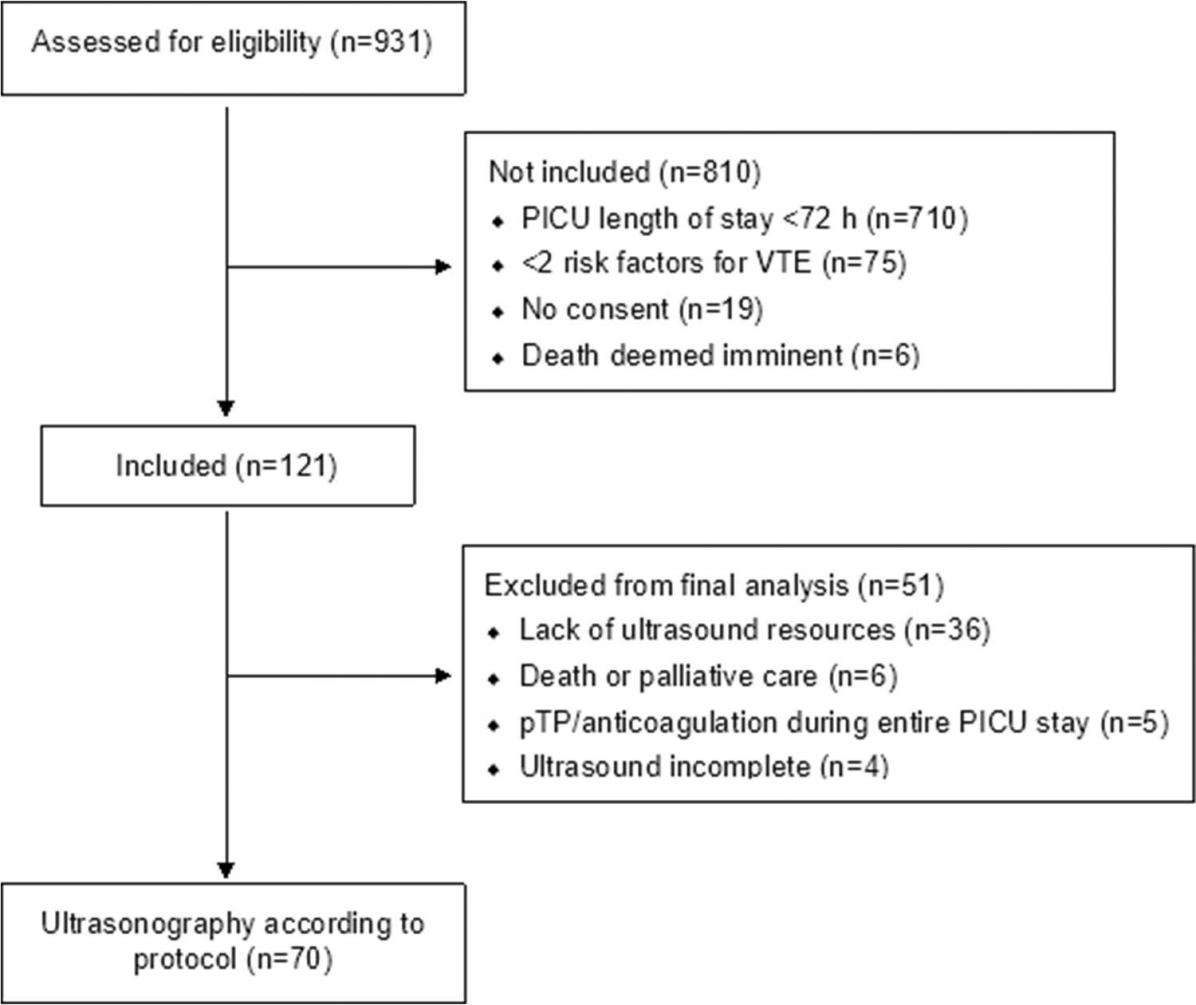
Flowchart showing selection of study patients. PICU, paediatric intensive care unit; VTE, venous thromboembolism; pTP, pharmacological thromboprophylaxis

During the study period, 146 ICU admission met the study inclusion criteria. One hundred twenty-one of these were included in the study (Fig. 1). Ultrasound screening was not performed in 42 patients. In 36 cases, this was due to lack of ultrasound resources and in 6 cases due to PICU mortality or limitation of care orders. Causes of death were severe ischemic brain injury (*n* = 2), necrotizing enterocolitis with bowel gangrene (*n* = 1), and vein of Galen malformation with severe heart failure (*n* = 1). In two patients, limitation of care decisions was made during their PICU stay, before transfer to a general ward. Both children suffered from rare congenital syndromes with short predicted life-span. Five patients (aged 8 weeks–17.7 years) were excluded since they received pTP or anticoagulation therapy. Two patients received prophylactic LMWH, one postoperatively and one following severe trauma. One patient received LMWH during the entire PICU stay due to Lemierre’s syndrome with internal jugular vein thrombosis and one patient due to CVC-related VTE. One patient was on CRRT and received a heparin infusion as anticoagulation. In four cases, the ultrasound evaluation could not be completed due to lack of patient cooperation or technical problems. Characteristics of included patients without ultrasound screening are supplied in supplemental Table S1. Seventy admissions were included in the final analysis (Fig. 1).

The median (IQR) patient age and weight were 0.3 (0–4.3) years and 5.2 (3.3–15.1) kg, respectively (Table 1). Forty-six (65.7%) patients were < 1 year of age, and 31 (44.3%) were female (Table 1). The most common admission diagnosis was respiratory failure, followed by surgery for congenital abnormalities, sepsis and seizures (Table 1). The median admission PIM-2 score was 4.6 (1.4–10.8). The median number of risk factors for VTE was 3 (2–4) (Table 2). Most patients (85.7%) needed a CVC during their PICU stay. Mechanical ventilation was required in 51 (72.9%) cases, with a duration of 8 (4–14) days. During the PICU stay, 28 patients fulfilled the criteria for sepsis. Two patients had previously had a VTE, and 1 patient suffered from a disease with increased risk of VTE (carbohydrate-deficient glycoprotein syndrome type Ia) (Table 2). Four (5.7%) patients needed CRRT for a median of 6.5 days (Table 1), three of them received anticoagulation with heparin during CRRT. Six patients (8.6%) required ECMO treatment. Median length of PICU and hospital length of stay were 9 (5–17) days and 22 (11–50.2) days, respectively (Table 1). Coagulation parameters at admission and discharge are shown in Table 3. During PICU stay, platelet count, antithrombin III, fibrinogen and d-dimer levels increased significantly, while INR and aPTT decreased.

**Table 1:** Characteristics of patients admitted to the paediatric intensive care unit for ≥ 72 h and with ≥ 2 risk factors for venous thromboembolism

**Variable**	**All patients** **(*n* = 70)**
**Age, years**	0.3 (0–4.3)
**Neonates, age < 1 month**	24 (34.3)
**Infants, age 1**–**12 months**	18 (25.7)
**Children, age 1**–**5 years**	14 (20)
**Children, age 5**–**12 years**	8 (11.4)
**Children, age > 12 years**	6 (8.6)
**Weight, kg**	5.2 (3.3–15.1)
**Gender, female**	31 (44.3)
**Prematurity (if age < 1 year)**	15 (21.4)
**Cause for PICU admission**	
Respiratory failure	16 (22.9)
Neonatal surgery	13 (18.6)
Sepsis	9 (12.9)
Seizures	6 (8.6)
Cardiac arrest	4 (5.7)
Cancer	3 (4.3)
Trauma	3 (4.3)
Other	16 (22.9)
**PIM-2, PDR**	4.6 (1.4–10.8)
**Mechanical ventilation, days**	8 (4–14)
**Continuous renal replacement therapy**	4 (5.7)
**Continuous renal replacement therapy, days**	6.5 (4–11.3)
**ECMO**	6 (8.6)
**PICU length of stay, days**	9 (5–17)
**Hospital length of stay, days**	22 (11–50.2)

All values are given as numbers (%) or as median (interquartile range)

*PICU* paediatric intensive care unit, *PIM-2* pediatric index of mortality score-3, *PDR* predicted death rate (%), *ECMO* extracorporeal membrane oxygenation

**Table 2:** Risk factors for venous thromboembolism in children admitted to the paediatric intensive care unit for ≥ 72 h

**Variable**	**All patients** **(*n* = 70)**
**Total number of risk factors**	3 (2–4)
**Central venous catheter**	60 (85.7)
**Mechanical ventilation**	51 (72.9)
**Age < 1 years**	42 (60)
**Surgical procedure during PICU stay**	33 (47.1)
**Sepsis**	28 (40)
**Renal failure**	11 (15.7)
**Congenital heart disease**	7 (10)
**Age > 12 years**	6 (8.6)
**Cancer**	5 (7.1)
**Trauma**	3 (4.3)
**Previous VTE**	2 (2.9)
**Genetic predisposition to VTE**	1 (1.4)

All values are given as numbers (%) or as median (interquartile range)

*PICU* paediatric intensive care unit, *VTE* venous thromboembolism

**Table 3:** Coagulation parameters and platelet counts of patients admitted to the paediatric intensive care unit for ≥ 72 h and with ≥ 2 risk factors for venous thromboembolism

**Variable**	**Admission**	***n***	**Discharge**	***n***	***P* value**
**INR**	1.4 (1.2–1.7)	51	1.2 (1.1–1.4)	47	< 0.01
**aPTT, s**	42 (36–51)	51	37 (33–44)	47	< 0.01
**AT III, kIE/L**	0.67 (0.46–0.98)	49	0.84 (0.54–1.18)	43	< 0.01
**Fibrinogen, g/L**	2.5 (1.6–3.2)	50	3 (2.3–3.8)	43	0.01
**d-dimer, mg/L**	0.87 (0.45–3.2)	49	2.5 (1–5.2)	43	0.048
**Platelet count, × 10**^**9**^**/L**	238 (145–347)	64	330 (191–454)	50	< 0.01

All values are given as median (interquartile range). *P* values between admission and discharge were obtained using Wilcoxon signed-rank test

*INR* international normalized ratio, *aPTT* activated partial thromboplastin time, *AT* antithrombin

Regarding the primary outcome, VTEs not related to a vascular catheter, no patient experienced a symptomatic VTE during their PICU stay and no VTE was found with US screening at PICU discharge. The resulting VTE incidence was 0% (95% CI: 0–5.1%). CVC-related VTE was diagnosed in 8 (11.4%, 95% CI: 4–19%) patients during their PICU stay. No cases of severe bleeding were found. In 46 patients, ultrasound screening could not be performed due to lack of ultrasound resources, incomplete ultrasound or palliation/death. Patient characteristics for these 46 patients are shown in supplemental Table S1. None of these patients developed a symptomatic VTE. This resulted in an incidence of symptomatic VTE not related to a vascular catheter of 0% (95% CI: 0–3.1%), considering all the 116 patients that were followed for signs of symptomatic VTE.

## Discussion

The main goal of this study was to obtain new data describing the incidence of VTE in a group of PICU patients considered to be at high risk of VTE. We used an extensive ultrasound screening programme to investigate the risk of VTE in 70 severely ill small children with multiple risk factors for VTE. VTE not related to the use of a CVC was chosen as the primary outcome of the study. The non-CVC associated VTE incidence of 0% (95% CI: 0–5.1%) found in our study indicates that VTEs not related to vascular catheters are an uncommon event even in a selected group of PICU patients with a median of three risk factors for VTE. Ultrasound screening was not performed in 46 patients due to lack of ultrasound resources, incomplete ultrasound or palliative care/death. However, none of these patients had a symptomatic VTE, resulting in an incidence of symptomatic non-CVC associated VTE of 0% (0–3.1%) in 116 patients.

Even though paediatric VTE is an uncommon event [14], severe illness necessitating PICU admission is considered to be a risk factor for VTE in children [15]. pTP is still usually not prescribed to PICU patients, even though there is a large variation in practice [7, 8]. This is most likely due to the low incidence of VTE described in the general PICU population [5]. However, previous studies on the incidence of VTE in the PICU are mainly retrospective or register-based, not including asymptomatic VTE. This carries the risk of underestimating the true incidence of VTE. There is a lack of prospective high quality data on the incidence of VTE in PICU patients, and there are currently no evidence-based guidelines regarding VTE prophylaxis in the PICU population. Considering the low incidence of DVT in children, experts have recommended that specific high risk patient populations should be targeted for VTE research [8, 16].

When prescribing pTP, the potential for patient benefit in terms of preventing a VTE must be weighed against definitive (pain and discomfort) and potential (bleeding, heparin-induced thrombocytopenia and neonatal osteopenia) side-effects. Our study provides new data helping the PICU physician to better assess this risk–benefit ratio for pTP in critically ill prepubertal children. This study also provides data useful for power calculation of RCTs evaluating the use of pTP in PICU patients.

From recent studies on paediatric CVC-related VTE, we have learned that the majority of CVC-related VTEs in children are asymptomatic [17]. Whether or not this is true also for other types of VTEs in the PICU has not previously been studied. Clinical diagnosis of VTE can be difficult in the PICU [16]. Small children are non-verbal, and sedation and mechanical ventilation often prevent communication of symptoms in older children. Also, symptoms can be ascribed to the underlying condition rather than to a VTE. Portal and renal vein thrombosis often present with non-specific symptoms and can be challenging conditions to identify [2]. Our results indicate that asymptomatic non-CVC associated VTE is rare in PICU-patients considered to be at high risk of VTE.

The primary outcome of this study was VTEs not associated with a CVC. There are several reasons for this. The incidence of and risk factors for CVC-related VTE in children have previously been extensively studied [18–21], whereas the previous data on VTEs not related to a vascular catheter are very sparse. The pathophysiology of CVC-related VTE has several distinct features, including endothelial damage, partial occlusion of the vein, and the presence of foreign material [22]. Also, the effectiveness of LMWH or other pTPs in preventing CVC-related VTE in children or adults is uncertain [22–26].

One of the strengths of our study is that we provide prospective data on the incidence of VTE in PICU patients, including a thorough screening for asymptomatic VTEs. Moreover, we targeted a PICU population considered to be at high risk of VTE. The comprehensive ultrasound screening was performed by experienced paediatric radiologists and sonographers, thereby avoiding underestimation of the true incidence of VTE. By evaluating upper extremity, lower extremity and abdominal veins, we included the majority of veins previously described to be at risk for VTE in children. In the study by Raffini et al. 19.5% of VTEs occurred in the portal, renal or caval veins [14], highlighting the importance of including these veins in US screening. The majority of patients in our study were neonates and infants, making our data representative for a group of patients where the coagulation system is not yet fully matured [27].

There are certain limitations to be considered when interpretating the results. This is a single-centre study from a general, tertiary paediatric ICU and the study population consisted mainly of small, prepubertal children. Our results may not be applicable to other centres or age groups, in particular older, postpubertal children. A larger patient sample would have given a more precise estimation of the true incidence of VTE in this group. The US screening procedure is very resource demanding and not always available, which led to a substantial loss to follow-up. This is a limitation of the study and the possibility of selection bias must be considered when interpreting the results. Moreover, we only included patients considered to be at high risk of VTE, thereby limiting the number of patients possible to include. A limitation of US is the lower sensitivity for VTE in veins where compressibility is difficult to assess [28]. However, US is an accepted screening method for VTE diagnosis [29]. In 6 patients included in the study, ultrasound evaluation could not be performed due to PICU mortality or limitation of care decisions. All these patients suffered from severe medical conditions explaining their deterioration, and none had clinical signs or suspicion of thromboembolic complications. Autopsy was performed in both patients with limitation of care orders and in one patient who died in the PICU due to necrotizing enterocolitis, and no VTEs were found in these patients.

## Conclusion

Using a comprehensive prospective screening programme in a group of critically ill children with a median of three risk factors of VTE, we did not find any VTEs that were not related to a CVC. Our results indicate that non-CVC associated VTE is an uncommon event even in a selected group of severely ill small children considered to be at high risk of VTE.

## Supplementary information

Below is the link to the electronic supplementary material.

Supplementary file1 (DOCX 15 KB)

## Abbreviations

APTT: Activated partial thromboplastin time
CRRT: Continuous renal replacement therapy
CVC: Central venous catheter
ECMO: Extracorporeal membrane oxygenation
INR: International normalized ratio
IQR: Interquartile range
LMWH: Low molecular weight heparin
PICU: Paediatric intensive care unit
PIM-2: Pediatric Index of Mortality-2
pTP: Pharmacological thromboprophylaxis
SD: Standard deviation
US: Ultrasound
VTE: Venous thromboembolism
