# Supplementary material for: Incidence of venous thromboembolic events not related to vascular catheters in a prospective cohort of critically ill children
Source: Eur J Pediatr. 2022 Jun 2;181(8):3031–8. doi: 10.1007/s00431-022-04487-8 (PMC9352609; doi:10.1007/s00431-022-04487-8)
Supplement: Supplementary file 1 — Supplementary file1 (DOCX 15 KB) [file 431_2022_4487_MOESM1_ESM.docx]

**Supplemental Table S1.** Characteristics of included patients without ultrasound screening.

| **Variable** | **All patients (n=46)** |
| --- | --- |
| **Age, years** | 0.4 (0.1-1.8) |
| **Neonates, age <1month** | 12 (26.1) |
| **Infants, age 1-12 months** | 17 (36.9) |
| **Children, age 1-5 years** | 12 (26.1) |
| **Children, age >5 years** | 5 (10.9) |
| **Weight, kg** | 5.8 (3.8-11.6) |
| **Gender, female** | 25 (54.3) |
| **Prematurity (if age <1 year)** | 8 (17.4) |
| **Cause for PICU admission** - Respiratory failure - Neonatal surgery  - Sepsis  - Seizures  - Cardiac arrest  - Cancer  - Trauma  - Other | 13 (28.3)  9 (19.6)  9 (19.6)  2 (4.3)  0 (0)  2 (4.3)  0 (0)  11 (23.9) |
| **PIM-2, PDR (n=43)** | 5.1 (1.3-11.6) |
| **Total number of risk factors** | 4 (3-5) |
| **Mechanical ventilation, days** | 8 (5-15) |
| **Continuous renal replacement therapy** | 7 (15.2) |
| **Continuous renal replacement therapy, days** | 10 (3-20) |
| **ECMO** | 4 (8.7) |
| **PICU length of stay, days** | 10 (7-20) |
| **Hospital length of stay, days** | 31 (12.5-57.8) |

All values are given as numbers (%) or as median (interquartile range). PICU, paediatric intensive care unit, PIM-2 pediatric index of mortality score-3, PDR predicted death rate (%), ECMO extracorporeal membrane oxygenation.
